# Valproic acid-induced amphiregulin secretion confers resistance to temozolomide treatment in human glioma cells

**DOI:** 10.1186/s12885-019-5843-6

**Published:** 2019-08-01

**Authors:** Jui-Chieh Chen, I-Neng Lee, Cheng Huang, Yu-Ping Wu, Chiu-Yen Chung, Ming-Hsueh Lee, Martin Hsiu-Chu Lin, Jen-Tsung Yang

**Affiliations:** 1 Department of Biochemical Science and Technology, National Chiayi University, Chiayi City, 60004 Taiwan; 2 Department of Medical Research, Chang Gung Memorial Hospital, Chiayi, 61363 Taiwan; 3 Department of Biotechnology and Laboratory Science in Medicine, National Yang-Ming University, Taipei, Taiwan; 4 Department of Earth and Life Sciences, University of Taipei, Taipei, Taiwan; 5 Department of Neurosurgery, Chang Gung Memorial Hospital, Chiayi, 61363 Taiwan; 6 College of Medicine, Chang Gung University, Tao-Yuan, 33302 Taiwan

## Abstract

**Background:**

Glioblastoma multiforme (GBM) is the most severe type of primary brain tumor with a high mortality rate. Although extensive treatments for GBM, including resection, irradiation, chemotherapy and immunotherapy, have been tried, the prognosis is still poor. Temozolomide (TMZ), an alkylating agent, is a front-line chemotherapeutic drug for the clinical treatment of GBM; however, its effects are very limited because of the chemoresistance. Valproic acid (VPA), an antiepileptic agent with histone deacetylase inhibitor activity, has been shown to have synergistic effects with TMZ against GBM. The mechanism of action of VPA on TMZ combination therapy is still unclear. Accumulating evidence has shown that secreted proteins are responsible for the cross talking among cells in the tumor microenvironment, which may play a critical role in the regulation of drug responses.

**Methods:**

To understand the effect of VPA on secreted proteins in GBM cells, we first used the antibody array to analyze the cell culture supernatant from VPA-treated and untreated GBM cells. The results were further confirmed by lentivirus-mediated knockdown and exogenous recombinant administration.

**Results:**

Our results showed that amphiregulin (AR) was highly secreted in VPA-treated cells. Knockdown of AR can sensitize GBM cells to TMZ. Furthermore, pretreatment of exogenous recombinant AR significantly increased EGFR activation and conferred resistance to TMZ. To further verify the effect of AR on TMZ resistance, cells pre-treated with AR neutralizing antibody markedly increased sensitivity to TMZ. In addition, we also observed that the expression of AR was positively correlated with the resistance of TMZ in different GBM cell lines.

**Conclusions:**

The present study aimed to identify the secreted proteins that contribute to the modulation of drug response. Understanding the full set of secreted proteins present in glial cells might help reveal potential therapeutic opportunities. The results indicated that AR may potentially serve as biomarker and therapeutic approach for chemotherapy regimens in GBM.

## Background

Neurons and glial cells are 2 major types of cells in the central nervous system (CNS) [1]. Glioma, a type of tumor that originates from glial cells, is usually found in the brain and occasionally in the spinal cord. Astrocytoma is the most common type of glioma in both adults and children. The World Health Organization (WHO) has assigned 4 grades to astrocytoma: pilocytic astrocytoma (Grade I), diffuse astrocytoma (Grade II), anaplastic astrocytoma (Grade III), and glioblastoma (Grade IV) [2, 3]. Glioblastoma, also known as glioblastoma multiforme (GBM), is the most aggressive and frequently diagnosed primary brain neoplasm. To date, surgical resection and radiotherapy, combined with adjuvant chemotherapy, are standard strategies for treatment of glioblastoma. The median survival of glioblastoma patients is only 12–15 months from diagnosis [4–6].

Temozolomide (TMZ), an oral alkylating agent, is regarded as the standard adjuvant chemotherapy due to its ability to cross the blood brain barrier (BBB) [7]. TMZ exerts its chemotherapeutic effect by methylation of the O^6^ position of guanine in DNA, leading to mispairing of O^6^-methylguanine with thymine. The futile repair of this base mismatch by the mismatch repair system causes single- and double-strand DNA breaks, resulting in cell cycle arrest and ultimately cell death [8]. Although most patients often show a dramatic initial response to TMZ, the overall response rate to TMZ-based chemotherapy still remains modest because of the development of drug resistance [9–11]. Therefore, the development of a novel combination strategy is urgently needed to reinforce the effectiveness of TMZ against GBM.

Although valproic acid (VPA) is widely used in the treatment of epilepsy, the pharmacological mechanisms are not fully understood. VPA may have anticonvulsant properties, as demonstrated by its increasing of γ-aminobutyric acid levels in the brain or by altering the properties of voltage-dependent sodium channels [12]. VPA is also a histone deacetylase inhibitor and is being evaluated as a treatment for various cancers [13, 14]. An accumulating body of evidence suggests that VPA combined with TMZ may enhance the antitumor effect of TMZ and increase the overall survival of patients with GBM [15–19]. However, the combination of TMZ and VPA is only slightly effective compared to the treatment of TMZ alone. The mechanism of anti-cancer action of VPA is still unclear. We explored the mechanism of action of VPA and attempted to find the novel target that enhances its anti-cancer effects. Combination therapy is an emerging treatment modality that combines two or more drugs to enhance therapeutic effects and improve patient survival rates.

Proteins secreted, shed or leaking from cells are collectively termed the secretomes [20]. Glial cells are capable of secreting a diverse quantity of secreted proteins that play pivotal roles in the physiology and pathology of the CNS [21–25]. In recent years, therapy-induced tumor secretomes have emerged as important candidate targets for the diagnosis and treatment of cancer [26–28]. To identify the secreted factors that contribute to the modulation of drug response, we used antibody array technology to screen the culture medium following VPA treatment. For the increased secretion of proteins caused by VPA treatment, we further analyze whether these may participate in drug resistance. Understanding the full set of secreted proteins present in glial cells might help reveal potential therapeutic opportunities.

In the results obtained from the antibody array experiments, we found that cells stimulated with VPA can promote the secretion of amphiregulin (AR) into the medium. AR is one of the ligands of the epidermal growth factor receptor (EGFR). Pro-AR is a transmembrane precursor that undergoes a series of proteolytic cleavage to release the mature soluble form. A previous study has indicated that AR was highly overexpressed in drug-resistant glioma cells. Silencing of AR has also been shown to enhance the drug sensitivity in resistant glioma cells by in vitro and in vivo experimental models [29]. More recently, a study further demonstrated that upregulated AREG mRNA level was characteristic for GBM tissue [30]. However, the effects of AR on TMZ treatment in GBM cells remains unknown. In this study, we demonstrate that TMZ resistance can be reversed by targeting AR in GBM cells.

## Methods

### Cell lines, reagents and chemicals

The human glioblastoma cell line U87MG, DBTRG-05MG, and M059K were obtained from the Bioresource Collection and Research Center (Food Industry Research and Development Institute, Hsinchu, Taiwan). TMZ-sensitive U87MG cells were unexpectedly obtained during long-term culture. Minimum Essential Medium (MEM) and fetal bovine serum (FBS) were purchased from Thermo Fisher Scientific (Waltham, MA, USA). Primary antibodies against cleaved PARP, cleaved caspase-3, and α-tubulin were obtained from Cell Signaling Technology, Inc. (Danvers, MA, USA). The primary antibody against AR was obtained from Santa Cruz Biotechnology, Inc. (Dallas, TX, USA). Total extracted protein concentration was determined by Bradford assay and the reagents were obtained from Bio-Rad Laboratories, Inc. (Hercules, CA, USA). TMZ, VPA sodium salt, dimethyl sulfoxide (DMSO), Trypan Blue, and other chemicals were purchased from Sigma-Aldrich Corp. (St. Louis, MO, USA). Propidium iodide (PI) solution was available from BD biosciences (San Jose, CA, USA).

### Cell culture and drug preparation

U87MG, DBTRG-05MG, and M059K cells were cultured in 1 × MEM supplemented with 10% FBS, 2 mM L-glutamine, 1% penicillin G/streptomycin (100 units/mL and 100 μg/mL, respectively), and placed at 37 °C in a humidified atmosphere of 5% CO_2_. The stock solution of TMZ was prepared in DMSO, VPA in distilled water and the final concentrations were diluted in culture medium.

### Cytotoxicity assay

Cytotoxicity was determined using a XTT-based cell proliferation kit (Biological Industries; Kibbutz Beit Haemek, Israel). Briefly, cells were seeded at 1 × 10^4^/well in a flat bottom 96-well plate (Greiner Bio-One GmbH; Frickenhausen, Germany), and starved in serum-free MEM for 4 h before drug treatment. After treatment with the specified concentration of drug for the indicated time interval, cells were incubated with XTT reagent at a ratio of 50 μL reagent to 100 μL of medium for a further 2 h. The absorbance at 450 nm of the sample was measured by an EnSpire® multimode plate reader (PerkinElmer; Billerica, MA, USA) against a control medium as a blank. The non-specific absorbance at 690 nm was also recorded and subtracted from the 450 nm measurement in each sample well.

### Cell-cycle analysis

Cells were seeded in 6-well dishes (Corning Incorporated-Life Sciences; Durham, NC, USA). After indicated treatments, cells were harvested and fixed in 70% ethanol at 4 °C for 16 h. The cell suspensions (1 × 10^6^/sample) were washed with 1 × PBS, and then stained with 10 μL of PI solution (BD Biosciences; San Jose, CA, USA). All samples were incubated in the dark at room temperature for 30 min. The cell cycle distribution was analyzed using a BD FACSCanto™ flow cytometer (San Jose, CA, USA).

### Western blot analysis

Cells were harvested and lysed in 1 × RIPA buffer (25 mM Tris-HCl pH 7.6, 150 mM NaCl, 1% NP-40, 1% sodium deoxycholate, and 0.1% SDS) containing a protease inhibitor cocktail. The protein concentrations of each extracted protein sample were measured using Bio-Rad protein assay reagents. A total of 30 micrograms of each protein sample was subjected to SDS-polyacrylamide gel electrophoresis. After electrophoresis, the proteins were transferred onto a polyvinylidene fluoride (PVDF) membrane. The membranes were blocked with 5% skim milk and then incubated with specific primary antibody overnight. The antibody-probed membrane was then washed 6 times with 1 × PBST (0.05% Tween 20 in 1 × PBS). After washing, the appropriate horseradish peroxidase-labeled secondary antibodies were added to the membrane for 1 h, and then washed with 1 × PBST. The bound antibodies were detected by enhanced chemiluminescence (ECL) reagents (GE Healthcare Life Sciences; Uppsala, Sweden). The blot signals were visualized by X-ray film (Roche Applied Science, Mannheim, Germany). The intensities of signals were quantified by GeneTools software (SYNGEN, Cambridge, UK).

### Human growth factor antibody array

Cells were cultured in 150-mm culture dishes for 70 ~ 80% confluence. Cells were washed with 1 × PBS, and then they were replenished with 500 μM VPA in serum-free MEM and cultured for another 48 h. The conditioned media were collected and centrifuged at 2000×*g* for 10 min at 4 °C to remove any cell debris. The media were further concentrated using Amicon® Ultra 15 mL centrifugal filters with a 3 k Da cut-off (Millipore; Bedford, MA, USA). Protein concentrations were measured by a Bio-Rad protein assay reagent. A total of 200 microgram proteins were used to hybridize to a membrane-form antibody array containing 41 human growth factors (ab#134002; Abcam; Cambridge, MA, USA).

### Lentivirus infection and shRNA knockdown for amphiregulin

The pLKO.1-puro-based lentiviral vectors, TRCN0000117298 (shAR#1), TRCN0000117995 (shAR#2), and pLKO.1-shScramble were obtained from National RNAi Core Facility at Academia Sinica, Taipei, Taiwan. Recombinant lentiviruses were packaged per the manufacturer’s instructions. Cultured cells were incubated with lentiviral supernatants supplemented with 8 μg/ml polybrene for 24 h, the medium was replaced fresh medium, and the cells were incubated for another 48 h. The stable cell lines were selected by puromycin (5 μg/ml).

### Human EGFR phosphorylation antibody array

U87MG cells were pre-starved in serum-free MEM for 4 h, and then they were either retained in serum-free MEM or treated with recombinant human AR proteins (R&D SYSTEMS; 50 ng/mL) for 10 min. A total of 200 microgram cell lysate were used to hybridize to a membrane-form antibody array containing 17 different phosphorylated human EGFRs (ab#134005; Abcam).

### Reverse transcription and real-time quantitative PCR

Total RNA was extracted using TRIzol reagent (MDBio Inc., Taipei, Taiwan). The mRNA was reverse transcribed into cDNA using M-MLV reverse transcriptase, Oligo (dT), and dNTP Mix (Promega, Madison, WI) as per manufacturer’s instructions. Synthesized cDNA (1 μg) was used as template for quantitative PCR (qPCR) that was conducted using KAPA SYBR FAST qPCR Kits (Kapa Biosystems, MA). The qPCR assays were performed in triplicate using iQ5 Optical System (Bio-Rad). The relative expressions of the gene were calculated using expression value. The cycle threshold (Ct) values were determined using iQ5 Optical System Software (Bio-Rad). The quantification of gene products was normalized to the expression of the glyceraldehyde-3-phosphate dehydrogenase (GAPDH) gene. The fold change was calculated as 2^-△△Ct^.

### In vivo xenograft tumor studies in athymic BALB/c mice

Suspensions of cultured wild type U87MG cells were injected subcutaneously into the left flanks of 5-week-old female athymic nude mice (CAnN.Cg-*Foxn1*^*nu*^/CrlBltw; BioLASCO Co., Ltd., Taiwan). The mice were kept in an AAALAC International (The Association for Assessment and Accreditation of Laboratory Animal Care International)-certificated animal center (Chang Gung Memorial Hospital, Chiayi, Taiwan) during the experiment with a temperature of 20–25 °C, relative humidity of 40–75% and a 12:12-h light-dark cycle, with free access to food and water. When volumes (length × width^2^ × 0.5) of tumors reached over 62.5 mm^3^, as measured by calipers, mice were randomly allocated into groups of four animals to receive TMZ (10 mg/kg/d), VPA (30 mg/kg), TMZ combined VPA and vehicle by intraperitoneal injection every other day. Tumor diameters of tumor-bearing mice were measured three times per week, and tumor volumes were calculated. When the experiment was terminated at the 21st day, mice were euthanized with an excess of CO_2_. The subcutaneous tumors were excised, weighed, and prepared for next analysis. The tumors were then fixed in 10% formalin, embedded in paraffin and subsequently processed for immunohistochemistry. All mice were handled in accordance with the Animal Care and Use Guidelines of the Chang Gung memorial hospital (Chiayi, Taiwan) under a protocol approved by the Institutional Animal Care and Use Committee. The AR knockdown U87MG cells (shAR) and shControl were also used in nude mice xenograft tumor studies. Each group has three animals, and received TMZ treatment only. The treatment and observation period was 2 weeks.

### Statistical analysis

Data are presented as the mean ± S.E.M. from at least 3 independent experiments. Statistical analysis was performed using the Student’s *t* test, which showed statistical significance when the *P* value was less than 0.05.

## Results

### Effects of combined TMZ and VPA on anti-cancer activity in U87MG cells

**Fig. 1:**
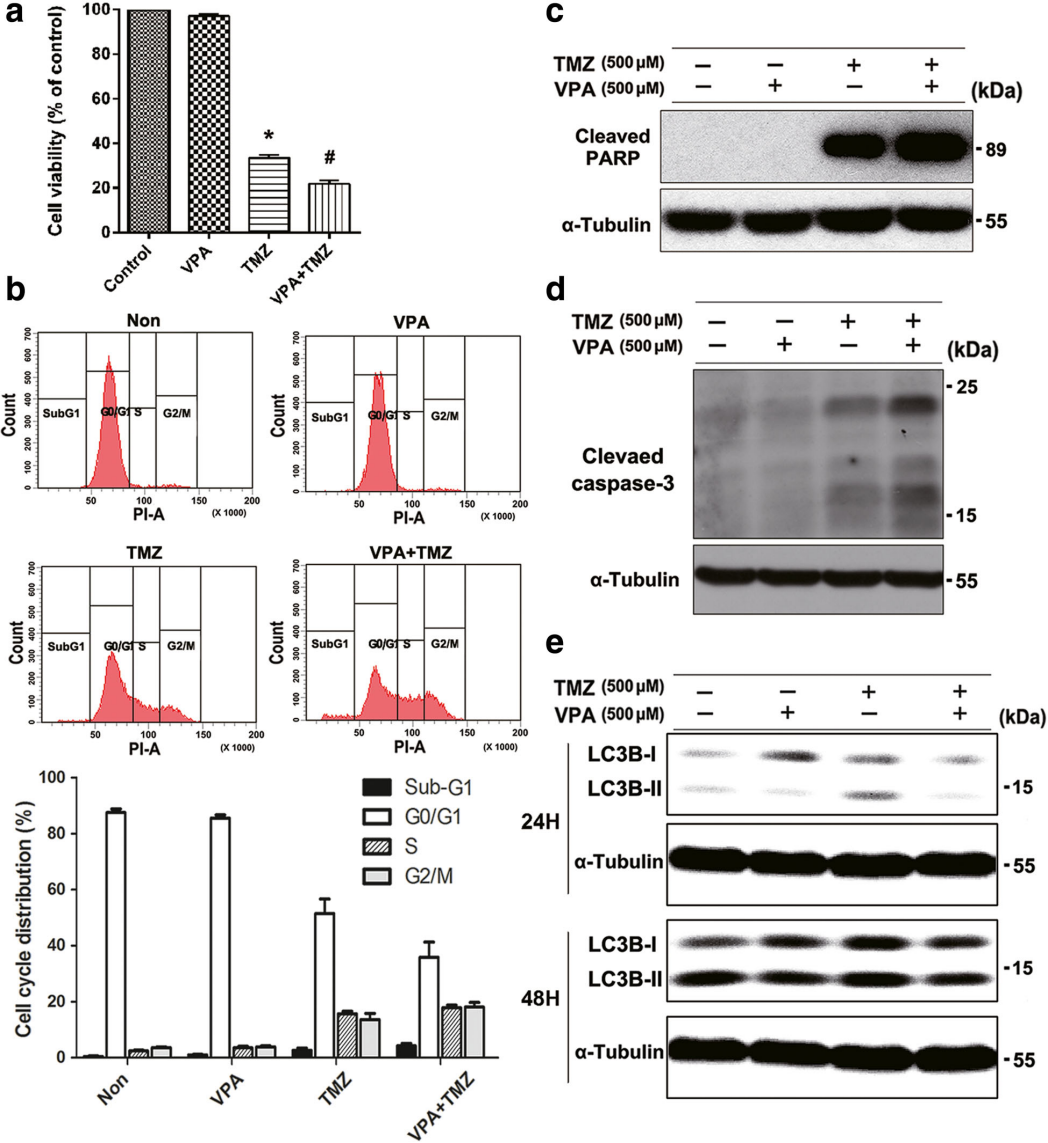
VPA regulated the cytotoxic effects of TMZ in U87MG cells. Cells were treated with VPA (500 μM) or TMZ (500 μM), alone or in combination, for 48 h. **a** Cell cytotoxicity was measured by an XTT-based assay. Data are presented as mean ± S.E.M. of at least 3 independent experiments. *P < 0.05, compared with the control (0 μM). #*P* < 0.05, compared with cells treated with TMZ (500 μM) alone. **b** Cell cycle was analyzed by PI staining and flow cytometry. Sub-G1, G0/G1, S and G2/M indicate different cell cycle phases (Upper panel). Bar graphs present the percentage of the number of cells in each phase. Data are presented as mean ± S.E.M. from 3 independent experiments (Lower panel). **c-e** The effects of VPA on TMZ-induced apoptosis and autophagy were assessed by Western blot using cleaved PARP, cleaved caspase-3, and LC3B antibodies

To evaluate the effect of combined VPA and TMZ on cell viability, cells were incubated in culture medium containing 500 μM VPA, 500 μM TMZ, or a combination of 500 μM VPA and 500 μM TMZ for 48 h. Combined treatment with TMZ and VPA significantly inhibited cell viability by about 12%, compared to that of TMZ alone (Fig. 1a). Next, the cell cycle distribution was determined by PI staining and flow cytometry. There was an increase in the sub-G1 peak from 2.6% in TMZ-treated cells to 4.2% in combined TMZ/VPA-treated cells (Fig. 1b). To understand the mechanisms by which VPA, TMZ, or TMZ/VPA combined treatment is related to the induction of apoptosis, cleaved PARP and cleaved caspase-3 were evaluated by Western blot analysis. The levels of cleaved PARP and cleaved caspase-3 were significantly higher with TMZ/VPA combined treatment than with TMZ alone (Fig. 1c-d). In addition, we also examined the expression of two microtubule-associated protein light chain 3 (LC3) forms, LC3-I and LC3-II, using Western blot analysis. During autophagy, LC3-I is converted to LC3-II, which is related to the extent of autophagosome formation. As shown in Fig. 1e, we detected an enhanced LC3-II band in TMZ-treated cells compared to untreated cells at 24 h. Following the 48 h treatment, we observed that VPA appears to reduce the extent of LC3-II. These results indicate that the addition of VPA results in an increase in the cytotoxic effect of TMZ against glioblastoma cells. Thus, it is essential to elucidate the precise mechanism underlying the effects of combined treatment in order to develop efficacious therapeutic regimens.

### AR was secreted into conditioned media in VPA-treated U87MG cells

**Fig. 2:**
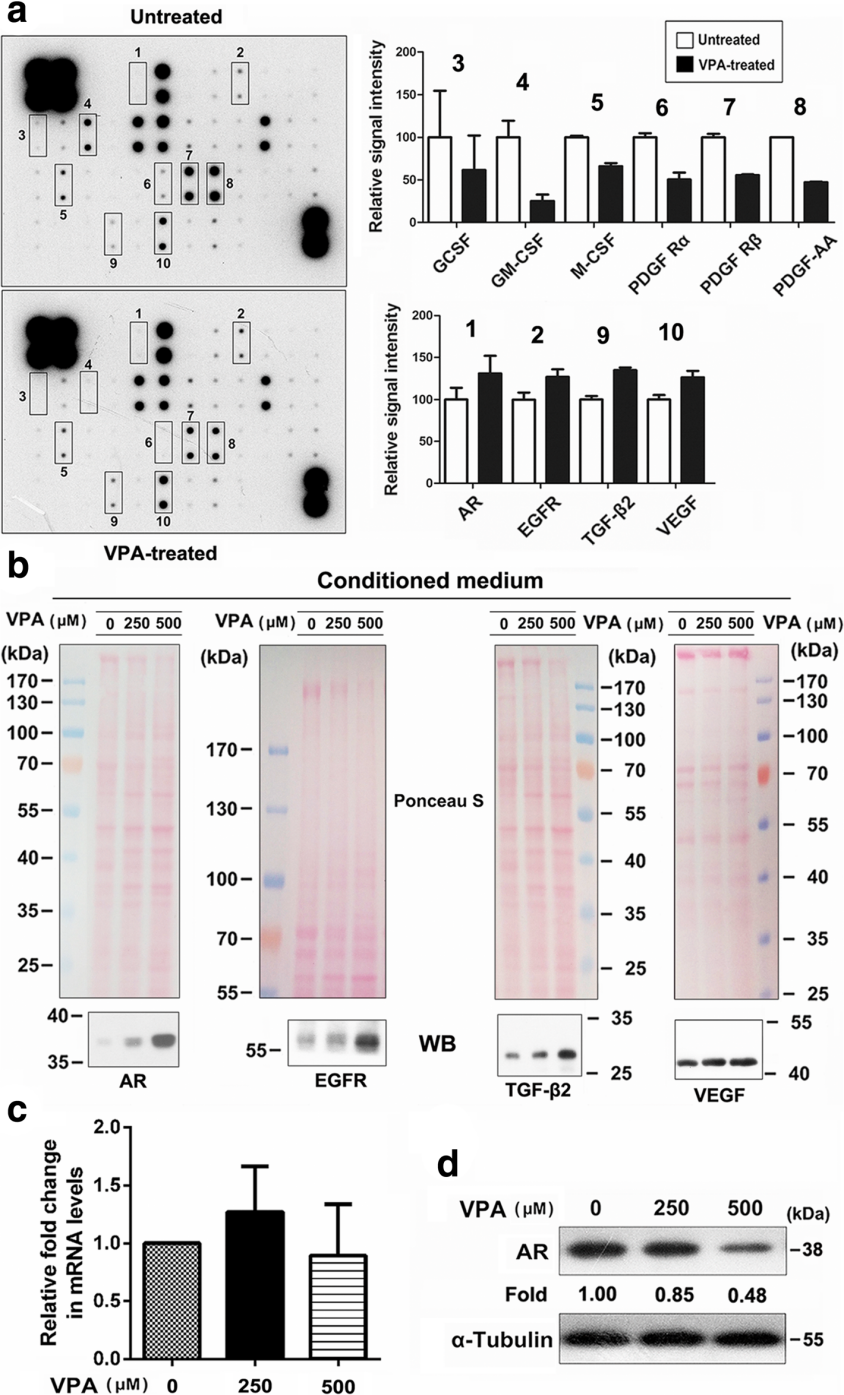
AR was secreted into the extracellular medium upon VPA treatment. **a** Cells were treated with or without 500 μM VPA for 48 h. The serum-free conditioned media were harvested as described in the Materials and Methods, and analyzed by a human growth factor antibody array. Left panel: Template shows the location of specific antibodies spotted on the membrane. Each antibody was spotted in duplicate. Right panel: The relative density of the spots was quantified by densitometry analysis. The histograms illustrate the relative amount of secretion proteins. **b** Secreted levels of AR, EGFR, TGF-2β, and VEGF were analyzed in cell culture media using Western blot. Equal protein loading was confirmed by membrane staining with Ponceau S. The results are representative of at least 3 independent experiments. **c-d** Cells were treated with the indicated concentrations of VPA (0–500 μM) for 48 h. The mRNA levels of AR in cells were measured by real-time PCR. Intracellular AR levels were analyzed in whole cell lysates using Western blot. α-Tubulin was used as a loading control

An accumulating body of evidence reveals that secreted growth factors play a potent role in modulating cancer progression and therapeutic response [26, 31]. To identify the secreted growth factors involved in VPA treatment in U87MG cells, we obtained conditioned media (CM) and conducted a human growth factor antibody array to screen a comprehensive secretion profile. In a comparison of secretion profiles in cells by stimulation with or without VPA, the growth factor antibody array could simultaneously screen 41 different molecules. As shown in Fig. 2a, the expression levels of 6 secreted proteins (GCSF, GM-CSF, M-CSF, PDGF Rα, PDGF Rβ, and PDGF-AA) were decreased and those of 4 secreted proteins (AR, EGFR, TGF-β2, and VEGF) were increased in VPA-treated cells, compared with the control. To further confirm the antibody array results, we performed Western blot to determine if AR, EGFR, TGF-β2, and VEGF were highly present in conditioned media of the VPA-treated U87-MG cells. As expected, VPA treatment resulted in significantly higher amounts of AR, EGFR, TGF-β2, and VEGF in conditioned media (Fig. 2b). The VPA-regulated profiles of protein secretion might exert a biological function that affects the sensitivity of TMZ treatment. Among the four proteins, the roles of AR in TMZ sensitivity have not been described in the literature. To prove the effect of VPA on AR levels, cells were cultured in the presence and absence of increasing concentrations of VPA for 48 h. As shown in Fig. 2c, VPA treatment did not cause a significant concentration-dependent increase in AR expression at the mRNA level. The relative levels of AR in cell lysates were analyzed by Western blot. The results showed that VPA decreased intracellular AR levels in a concentration-dependent manner (Fig. 2d). These data suggest that VPA can significantly increase the secretion of AR from the cells, which might be a critical regulator in VPA therapy and further affect the efficacy of combined VPA/TMZ treatments.

### AR might be involved in resistance to TMZ in U87MG cells through EGFR activation

**Fig. 3:**
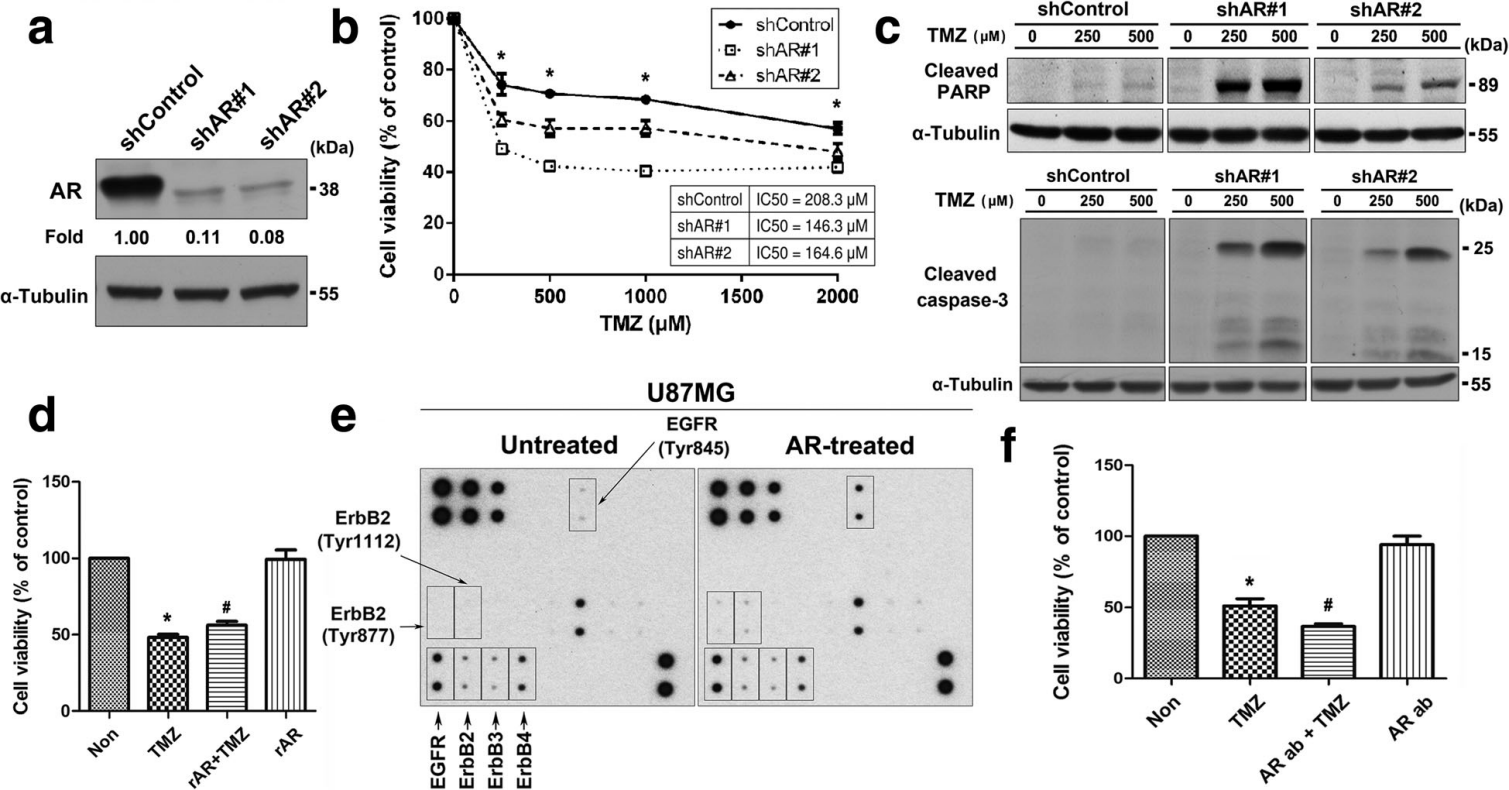
AR may confer resistance to TMZ in U87MG cells through EGFR activation. **a** The protein levels of AR in U87MG/shControl, U87MG/shAR#1, and U87MG/shAR#2 cells were examined using Western blot. **b** Cells were exposed to increasing concentrations of TMZ for 48 h, and subsequently cytotoxicity was evaluated by an XTT-based assay. The percentage of cell viability is shown relative to untreated controls. *P < 0.05 compared with shControl group. **c** The cell lysates were prepared, and equal amounts of total cell lysates were subjected to Western blot with the indicated antibodies (cleaved PARP and cleaved caspase-3). **d** Cells were pre-treated with rAR (50 ng/ml) for 8 h, and then further co-treated with TMZ (1500 μM) for 48 h. Cell viability was measured by XTT assay. **e** The effect of AR on the activation of EGFR family receptors was analyzed by a human EGFR phosphorylation antibody array. **f** Cells were pre-treated with AR antibodies (1 μg) for 2 h followed by TMZ (500 μM) treatment for another 48 h, and subsequently cytotoxicity was evaluated by an XTT-based assay. The percentage of cell viability is shown relative to untreated controls. *P < 0.05 compared with untreated control. #*P* < 0.05 compared with TMZ-treated group. Data are representative of at least 3 independent experiments performed in triplicate

To further delineate the role of AR in prompting resistance to TMZ in U87MG cells, we silenced AR expression using a lentivirus-mediated shRNA system. Compared to a control vector (shControl), AR was successfully knocked down in U87MG cells (Fig. 3a). Silencing AR expression in U87MG cell resulted in elevated sensitivity to TMZ in a dose-dependent fashion, thus suggesting that AR is a key regulator of TMZ resistance (Fig. 3b). We next investigated whether AR is involved in TMZ-induced apoptosis in U87MG cells. As shown in Fig. 3c, knockdown of AR markedly increased the TMZ-induced amount of cleaved PARP and cleaved caspase-3. To further clarify the effect of AR on TMZ sensitivity, cells were pre-treated with exogenous recombinant AR (rAR) to investigate whether AR can mediate TMZ sensitivity. The results revealed that pre-treatment with rAR significantly conferred increased resistance to TMZ (about 8%), compared with TMZ alone (Fig. 3d). To evaluate the effect of AR on the activation of EGFR family receptors, we used a human EGFR phosphorylation antibody array to analyze the phosphorylation levels of 17 different EGF Receptors. As shown in Fig. 3e, we detected significantly elevated levels of phospho-EGFR (Tyr845) in rAR-treated U87MG cells when compared with untreated cells. Moreover, we also observed a slight increase in ErbB2 phosphorylation on Tyr877 and Tyr1112 following rAR stimulation. To further verify the effect of AR on TMZ sensitivity, we conducted an antibody neutralization experiment. As shown in Fig. 3f, cells pre-treated with AR antibody markedly improved the sensitivity to TMZ. Taken together, these results suggest that the presence of AR within the tumor microenvironment probably promotes and sustains EGFR activation, leading to drug resistance. Depletion of AR might be an effective intervention strategy to improve GBM sensitivity to TMZ.

### Increased expression of AR is associated with increased resistance to TMZ in human glioblastoma cell lines

**Fig. 4:**
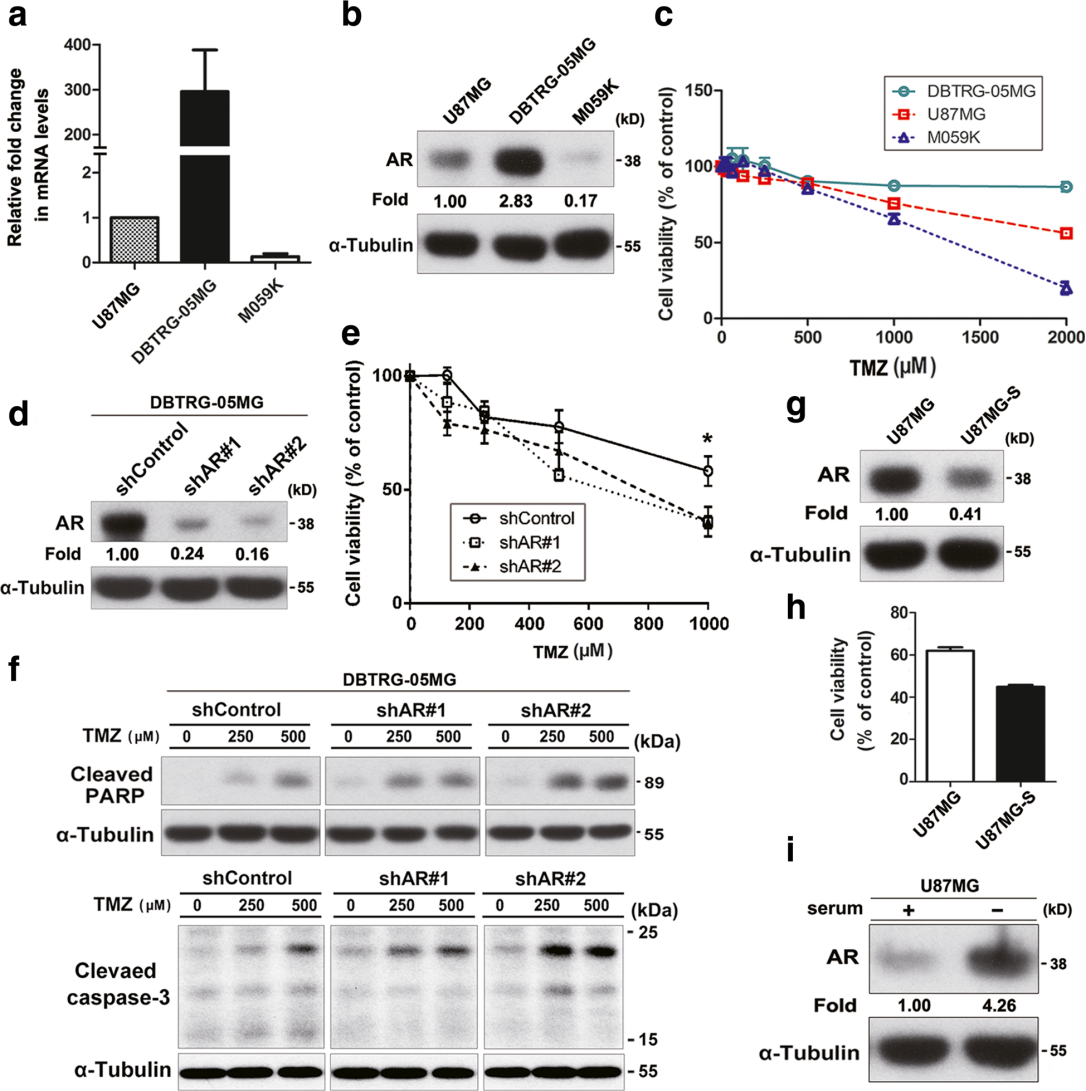
The expression level of AR was correlated with TMZ resistance in GBM cell lines. **a-b** The expression levels of AR in each GBM cell lines were evaluated by using quantitative real-time PCR and Western blot analysis. **c** Cells were treated with increasing concentrations of TMZ for 24 h, and then cell viability was determined by XTT assay. **d** The protein levels of AR in DBTRG-05MG/shControl, DBTRG-05MG/shAR#1, and DBTRG-05MG/shAR#2 cells were examined using Western blot. **e** Cells were exposed to increasing concentrations of TMZ for 48 h, and subsequently cytotoxicity was evaluated by an XTT-based assay. The percentage of cell viability is shown relative to untreated controls. *P < 0.05 compared with shControl group. **f** The cleaved PARP and cleaved caspase-3 were analyzed by Western blot. **g-h** The expression of AR in U87MG-S (TMZ-sensitive) and parental U87MG was evaluated by Western blot analysis. Cells treated with TMZ (1000 μM) for 24 h, and cell viability was measured by XTT assay. *P < 0.05 compared with U87MG cells. **i** U87MG cells were cultured in serum withdrawal medium for 24 h, and the effect of serum starvation on AR expression was analyzed by Western blot. Data are representative of at least 3 independent experiments performed in triplicate

To determine whether the expression of AR is associated with the sensitivity to TMZ in GBM cell line, we evaluated the expression of AR in three GBM cell lines (DBTRG-05MG, U87MG and M059K cells) by real-time quantitative PCR and Western blot (Fig. 4a-b). The results revealed that the DBTRG-05MG cell line exhibited the highest expression level of AR, followed by U87MG and M059K cells. According to the results of the XTT assay, DBTRG-05MG cells showed the highest resistance to TMZ, followed by U87MG and M059K (Fig. 4c). We then further suppressed AR expression in DBTRG-05MG cells and observed whether the sensitivity to TMZ could be increased. As shown in Fig. 4d-e, knockdown of AR led to increased sensitivity to TMZ in a dose-dependent fashion. In addition, knockdown of AR markedly exacerbated TMZ-induced apoptosis in DBTRG-05MG cells, as indicated by increases in cleaved PARP and cleaved caspase 3 (Fig. 4f). These data suggest that the levels of AR expression were significantly correlated with the sensitivity to TMZ in GBM cells. During the culture process, we found a mutated U87MG cell line (U87MG-S cells) that was more sensitive to TMZ than the original U87MG cells. Subsequently, we compared the relationship between AR expression and TMZ sensitivity in the two groups. As shown in Fig. 4g-h, U87MG-S cells, with lower basal levels of AR, exhibited the higher sensitivity to TMZ. Serum deprivation in cell culture mimics cellular stress, which has proven to be an effective strategy for sensitizing cancer cells to chemotherapeutic drugs [32, 33]. Therefore, we analyzed whether serum starvation can induce the expression of AR to adapt environmental changes, which might prevent cell death and make it more resistant to harsh environments, such as drug treatment. As shown in Fig. 4i, serum deprivation induced AR expression in U87MG cells. These results indicated the protective role of AR against TMZ treatment may cause GBM cells to develop drug resistance.

### Knockdown of AR enhances the sensitivity of human glioblastoma cells to TMZ in a mouse model

**Fig. 5:**
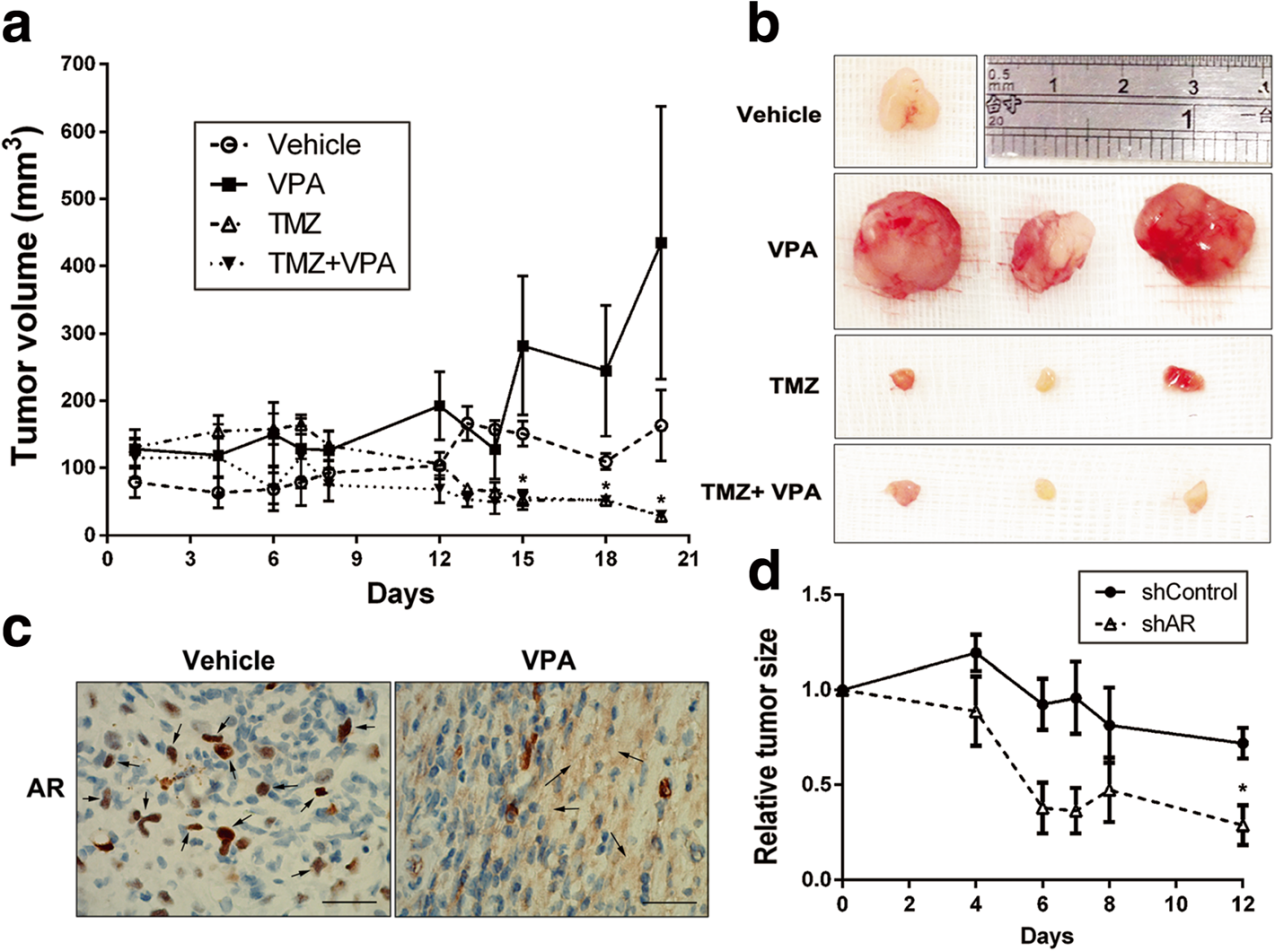
Reduction of AR expression increased chemosensitivity of glioma cells to TMZ in vivo. **a** U87-MG cells were inoculated subcutaneously into the flank of nude mice. Mice with appropriate size of tumors were divided randomly into four groups (*n* = 4) including vehicle-treated group, VPA-treated group (30 mg/day), TMZ-treated group (10 mg/kg/day) and combination of two. TMZ and VPA were administered by intraperitoneal injection once every 2 days for 20 days. **b** Photographs of xenograft tumors from each group were captured on day 21 after treatment. **c** AR expression in xenograft tumors in vehicle-treated and VPA-treated groups were analyzed by immunohistochemical staining with an antibody against AR (arrows indicate increased secretion). Scale bar = 100 μm. **d** AR knockdown enhanced response to TMZ treatment in vivo (*n* = 3)

We also further investigated the synergistic effects of a combination of VPA and TMZ in a human glioma xenograft-nude mouse model. In xenograft growth assay, the TMZ alone and TMZ/VPA significantly inhibited the growth of tumor cells compared to VPA alone. However, VPA did not cause any significantly synergistic effects on TMZ to reduce tumor cell growth in vivo (Fig. 5a). To verify these findings, we isolated tumor tissues and took images to visualize the tumor size. As shown in Fig. 5b, no significant differences were observed among TMZ alone and TMZ plus VPA. Next, tumor tissues from mice were further characterized by immunohistological (IHC) staining. The results suggested that VPA can significantly promote AR secretion in vivo (Fig. 5c). To investigate the in vivo effect of AR silencing on TMZ sensitivity, U87-MG cells transduced with either a shControl or AR shRNA were inoculated subcutaneously into the flank of nude mice. After the tumor cells were grown to a suitable size, the mice were given TMZ. As shown in Fig. 5d, knockdown of AR increased chemosensitivity of glioma cells to TMZ compared to shControl group.

## Discussion

GBM is the most common and most lethal type of primary brain tumor, and the 5-year relative survival of patients who are diagnosed with GBM is less than 5% [34]. Identification of a molecular pathogenesis could allow for the development of new therapeutics that target signaling pathways in malignant gliomas. However, only a limited number of chemotherapeutic drugs are available to glioma patients due to the tumor location and difficulty in permeating the BBB. An anti-epileptic drug like VPA can be used to relieve seizures in primary brain tumor patients. Our previous study indicated that VPA had the synergistic role with TMZ in anti-glioma cells. In the present study, our results showed that TMZ can induce autophagy activation, and when TMZ combined with VPA, VPA appears to reduce the degree of autophagy. Previous studies have shown that TMZ treatment can induce autophagy, allowing cancer cells to survive under adverse environmental conditions, thereby contributing to the development of drug resistance in glioma [35, 36]. Recently, the autophagy inhibitors have attracted attention as a potential agent to overcome TMZ-resistance [37–40]. Although TMZ enhanced the extent of autophagy in our data, the precise role of autophagy in the combination of VPA and TMZ for glioma cells is unclear and further research is needed.

Recent studies have indicated that secreted proteins play key roles in the control and regulation of numerous biological and disease processes, including cancer progression. To identify the influence of VPA treatment on the secreted proteins that contribute to TMZ sensitivity, a human growth factor antibody array-based screen of the culture medium was conducted. The results indicated that VPA-induced AR secretion confers resistance to TMZ treatment in U87MG cells.

VPA is a branched short-chain fatty acid derived from naturally occurring valeric acid. It has demonstrated neuroprotective effects against various insults through multiple cellular pathways. In addition, VPA has been shown to have anti-cancer effects against a wide variety of neoplasms [41–48]. The anti-tumor activity of VPA was reported to be attributed to its ability to inhibit histone deacetylase [13, 49]. Previous studies have also found that VPA influences cellular function by regulating protein secretion. In adipocytes, VPA is able to decrease leptin secretion, thus contributing to weight gain [50]. A previous study showed that VPA influences angiogenesis by down-regulating VEGF secretion in colon carcinoma cells [51]. Moreover, treating pancreatic and colon cancer cells with VPA leads to inhibition of tumor cell growth by down-regulation of β-amyloid precursor protein (APP) and secreted soluble APPα [52]. Another histone deacetylase inhibitor, suberoylanilide hydroxamic acid (SAHA), has anti-cancer effects through its ability to increase secretion of HSP60 via exosomes in human lung-derived carcinoma cells [53]. Another survey found that VPA exerts anti-tumor effects through inhibition of tumor–stromal interaction [54]. In the present study, for the first time, we examined the effect of VPA on secreted proteins in glioma cells.

AR, a ligand of EGFR, is synthesized as a transmembrane precursor that undergoes a series of proteolytic processes to yield a mature secreted form [55]. Accumulating evidence indicates that high-level expression of AR is associated with progression in various types of cancers, including colorectal cancer [56, 57], breast cancer [58, 59], ovarian cancer [60, 61], pancreatic cancer [62, 63], lung cancer [64, 65], liver cancer [66, 67], and oral cancer [68]. AR was reported to have oncogenic effects in many cancer cell types and was implicated in drug resistance [67]. Previous studies have indicated that AR is able to regulate the activation of extracellular signal-regulated kinase (ERK) through EGFR, leading to the progression of cancers [63, 69]. In addition, a previous study indicated that AR is significantly over-expressed in non-responders, but undetectable in responders, which may biologically affect drug sensitivity and lead to the resistance of non–small cell lung cancer (NSCLC) cells to EGFR tyrosine kinase inhibitors in vitro [70]. Another study found that increased serum AR levels were significantly correlated with an unfavorable response to EGFR tyrosine kinase inhibitors, by identifying patients with a higher probability of resistance to the drug [71]. AR expression in human glioma cells was reported to be associated with increased ERK activation, resulting in the resistance of glioma cells to cannabinoid treatment [50]. Moreover, in vivo silencing of AR rendered the xenografts of resistant tumors sensitive to cannabinoid antitumoral action [29].

Gene amplification of EGFR is frequently seen in GBM, and a minority of tumor cells in GBM carries an exon deletion form, the EGFR variant III (EGFRvIII). GBM with the EGFRvIII mutation is unable to bind ligands and is frequently highly aggressive. So far, inhibition of EGFR signaling with RTK inhibitors has not been satisfactory in clinical use; one of the reasons for treatment failure is the redundancy of RTK pathways.

TGF-β/Smads signaling is believed to be involved in cell proliferation, differentiation, invasion and metastasis [72]. The TGF-β gene superfamily is comprised at least 3 TGF-βs (β1, β2 and β3), which are correlated with different degrees of human glioma malignancy [73]. TGF-β is a multifunctional cytokine, and can be secreted by tumor cells and non-tumor cells, including immune cells and stromal cells within the microenvironment. Dysregulated signaling in glioblastoma cells mediated resistance to TGF-β-induced growth inhibition, or acquired proliferation ability in response to this cytokine, and even produced greater amounts of TGF-β. Targeting for TGF-β inhibition in glioma, including antisense oligonucleotide for TGF-β2, kinase inhibitor of type I TGF-β receptor and TGF-β antibodies, is currently at the clinical trial stage [74]. Angiogenesis is an obvious feature of glioblastoma, and targeting of VEGF-A ligand with bevacizumab, a humanized monoclonal antibody, has shown efficacy in recurrent glioblastoma. However, a large-scale clinical trial of bevacizumab did not improve overall survival in patients with newly diagnosed glioblastoma [75]. In our study, VPA slightly enhanced VEGF secretion in U87MG cells, in contrast to a previous study [76].

For the decreased expression of VPA-induced secreted growth factors, G-CSF is a glycoprotein that stimulates bone marrow to produce granulocytes and stem cells. Use of G-CSFs has been shown to reduce the incidence of febrile neutropenia when administered with chemotherapy. Upregulation of GM-CSF was found in both human and mouse glioma microenvironments, compared with normal brain or peripheral blood samples [77]. It was found that glioblastoma-derived M-CSF induces microglial cells to release insulin-like growth factor-binding protein 1 (IGFBP1) to promote angiogenesis [78]. Over-activity of PDGF signaling is linked to the development of glioblastoma, and increased expression of PDGF and PDGF receptors can also be found in pericytes of tumor vasculature and in the stromal fibroblasts and myofibroblasts that contribute tumorigenesis [79, 80]. In addition, high levels of PDGF drive mouse glial cells to differentiate into an oligodendrocyte lineage, which will develop into highly malignant oligodendroglial tumors [79]. In our data, VPA suppressed the expression of PDGF AA and PDGF Rβ, and might have a beneficial role to play.

## Conclusions

In summary, combining 2 or more different drugs in the development of novel therapeutic strategies is often adopted in cancer studies; however, the beneficial and adverse effects must be carefully analyzed, especially when the drugs have some unknown functions. In this study, we found changes in some secreted growth factors due to VPA treatment, and all of these differentially expressed growth factors were involved in glioma tumorigenesis. Knockdown expression of AR, one of the target proteins activated by VPA, would make glioma cells more sensitive to TMZ treatment. In this study, we can overcome the TMZ resistance by providing a more specific candidate target to improve malignant glioma therapeutics.

## Data Availability

All data used to support the findings of this study are included within the article. The original data and specific AR-knockdown U87MG cells used in this study are also available from the corresponding author upon request.

## Abbreviations

APP: Amyloid precursor protein
AR: Amphiregulin
BBB: Blood brain barrier
CM: Conditioned media
CNS: Central nervous system
DMSO: Dimethyl sulfoxide
EGFR: Epidermal growth factor receptor
ERK: Extracellular signal-regulated kinase
FBS: Fetal bovine serum
GAPDH: Glyceraldehyde-3-phosphate dehydrogenase
GBM: Glioblastoma multiforme
G-CSF: Granulocyte-colony stimulating factor
GM-CSF: granulocyte-macrophage colony stimulating factor
HSP: Heat shock protein
IGFBP1: Insulin-like growth factor-binding protein 1
IHC: Immunohistochemistry
LC3: Microtubule-associated protein light chain 3
M-CSF: Macrophage colony-stimulating factor
MEM: Minimum essential medium
NSCLC: Non–small cell lung cancer
PDGF: Platelet-derived growth factor
PI: Propidium iodide
PVDF: Psolyvinylidene fluoride
SAHA: Suberoylanilide hydroxamic acid
SDS: Sodium dodecyl sulfate
TGF-β: Transforming growth factor-beta
TMZ: Temozolomide
VEGF: Vascular endothelial growth factor
VPA: Valproic acid
WHO: World Health Organization
XT: 2,3-bis-(2-methoxy-4-nitro-5-sulfophenyl)-2H-tetrazolium-5-carboxanilide
